# Efficient Extraction from Mice Feces for NMR Metabolomics Measurements with Special Emphasis on SCFAs

**DOI:** 10.3390/metabo9030055

**Published:** 2019-03-21

**Authors:** Adrian Hauser, Philipp Eisenmann, Claudia Muhle-Goll, Burkhard Luy, Andreas Dötsch, Daniela Graf, Pavleta Tzvetkova

**Affiliations:** 1 Karlsruhe Institute of Technology, Institute of Organic Chemistry, Fritz-Haber Weg 6, 76131 Karlsruhe, Germany; adrian.hauser@partner.kit.edu (A.H.); philipp-michael.eisenmann@kit.edu (P.E.); burkhard.luy@kit.edu (B.L.); 2 Karlsruhe Institute of Technology, Institute for Biological Interfaces-4, Magnetic Resonance, Hermann-von-Helmholtz-Platz 1, 76344 Eggenstein-Leopoldshafen, Germany; claudia.muhle-goll@kit.edu; 3 Max Rubner-Institut, Haid-und-Neu-Straße 9, 76131 Karlsruhe, Germany; andreas.doetsch@mri.bund.de (A.D.); daniela.graf@mri.bund.de (D.G.)

**Keywords:** metabolomics, mice, feces, faeces, NMR spectroscopy, short chain fatty acids, dietary fibers, extraction protocol, diet, NMR, extraction

## Abstract

Nuclear magnetic resonance (NMR) spectroscopy is one of the most promising methods for use in metabolomics studies as it is able to perform non targeted measurement of metabolites in a quantitative and non-destructive way. Sample preparation of liquid samples like urine or blood serum is comparatively easy in NMR metabolomics, because mainly buffer and chemical shift reference substance are added. For solid samples like feces suitable extraction protocols need to be defined as initial step, where the exact protocol depends on sample type and features. Focusing on short chain fatty acids (SCFAs) in mice feces, we describe here a set of extraction protocols developed with the aim to suppress changes in metabolite composition within 24 h after extraction. Feces are obtained from mice fed on either standard rodent diet or high fat diet. The protocols presented in this manuscript are straightforward for application, and successfully minimize residual bacterial and enzymatic activities. Additionally, they are able to minimize the lipid background originating from the high fat diet.

## 1. Introduction

A certain lifestyle including a healthy diet is expected to be the key for long and healthy life. However, many people in industrialized countries do not adhere to this kind of lifestyle and in consequence suffer from metabolic disorders such as obesity, type II diabetes, and cardiovascular disease [1,2]. Understanding the mechanisms of food metabolism by the gut and its microbial inhabitants is a key to identify links between diet and health. The recent advances in metagenomics have underlined the importance of a healthy intestinal microbial community (the microbiota) for human health [3]. Certain food components are indigestible by the human intestine itself and thus are available for microbial fermentation, which in turn produces metabolites, including vitamins [4]. Mostly, plant-derived carbohydrates commonly termed ‘dietary fiber’ reach the large intestine where they serve as main substrate for microbial fermentation [5]. As reviewed recently [6], the resulting short chain fatty acids (SCFA)—especially acetate, propionate and butyrate—are readily taken up by the intestinal epithelium and serve as a main source of energy to the epithelial cells. Apart from that, SCFA may serve as signaling molecules affecting the body as a whole, e.g., by regulating satiety or interfering with the immune system [6].

The protocol set presented in this work has been developed for the analysis of metabolites from DIO mice fecal samples with a special focus on the quantification of SCFA due to their above proposed role as signaling molecules by nuclear magnetic resonance (NMR) spectroscopy. The mouse is an established model for the study of nutrition research and enables dietary intervention studies for prolonged time [7,8]. Mice fecal samples are easily accessible and thus can be used to investigate changes in the intestinal environment such as microbiota and metabolite profile throughout the whole study period. For obesity studies high fat diets (HFD) are frequently used to generate diet induced obesity (DIO) in mice.

Feces cannot be considered as uniform biological material. Bacteria comprise about 50% of the total solids in human feces [9], but similarly to other components, microbiota composition mirrors dietary as well as environmental or genetic effects. A number of publications has dealt with the impact of different fecal extraction methods, focusing on metabolite coverage and integrity, taking into consideration that bacterial activity and chemical degradation may affect sample stability (for review see [10]). The gastrointestinal tract contains large amounts of both host proteases and bacterially released proteases [11], parts of which are excreted in feces [12]. Yet, fecal protease activity has not been extensively addressed in fecal metabolomics to our knowledge.

**Figure 1:**
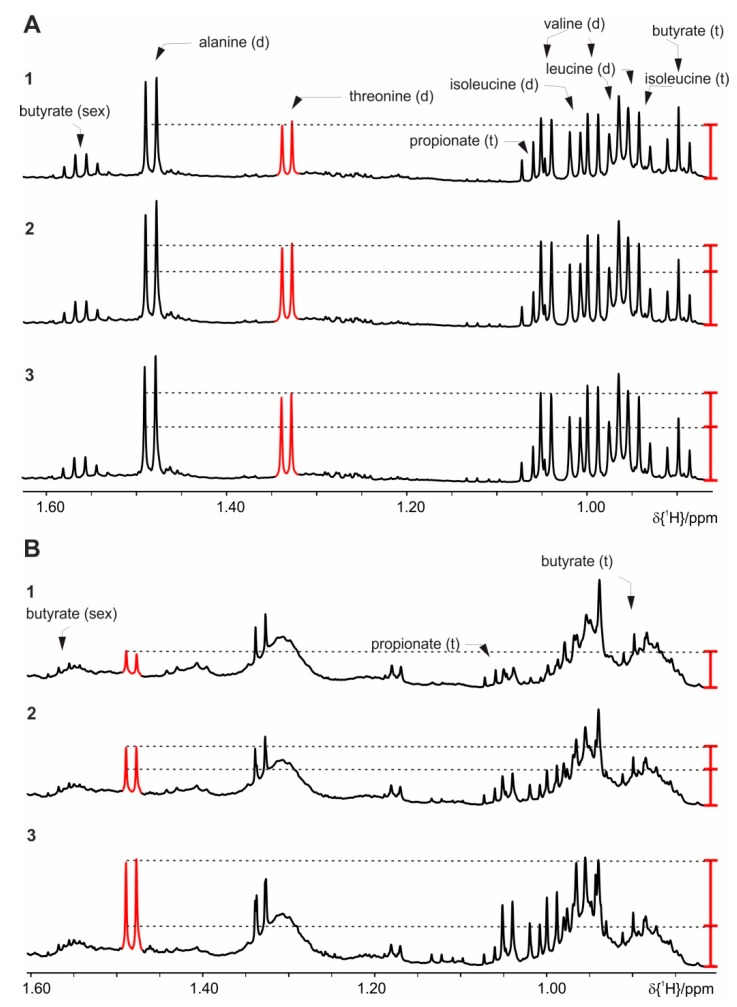
Proton nuclear magnetic resonance (NMR) spectra, which demonstrate changes in some of the metabolites extracted with phosphate buffer with extended storage at RT: (**A**) Mice fed on standard diet, (**B**) on high fat diet (HFD). **1** sample kept at 4 °C before measurement, **2** after 6 h, and **3** after 24 h in the sample changer at RT. For clarity only the spectral region of interest, harboring resonances of the short chain fatty acid (SCFA), is shown. The red bar indicates the increased signal intensities.

The majority of published NMR studies of feces typically uses phosphate buffer extraction, which allows the extraction of the majority of water soluble metabolites [13,14,15,16,17,18,19,20,21,22]. When we apply these protocols [13,14,15,16,17,18,19,20,21,22,23], we face sample stability problems over time, which were not reported in the original publications. Suppression of bacterial activity by sodium azide does not solve the problem. Thus we suspect strong enzymatic activity. Several approaches to modify the phosphate buffer protocol to overcome this problem have been tried, but none of them has been sufficient (Figure 1). Employing a mixed organic solvent with saline buffer finally solves the stability problem, however, at the expense of an increased lipid background, which prevented the detection and proper integration of the targeted SCFA in the spectra (as demonstrated in the Results Section in Figure 6). 

Before describing the protocol, which addresses the above problems, specific NMR related aspects have to be explained: NMR spectroscopy generally requires the use of deuterated (99.9%) solvents as any protonated compound will strongly contribute to the signals in the spectra and could potentially overlap with metabolite signals. Despite the fact that deuterated solvents have nowadays considerably reduced prices, common (cheap) solvents are preferred for protocols suitable for large studies. Thus, feces could be initially extracted with a non-deuterated solvent that is then replaced (e.g., by lyophilisation) by an NMR compatible solvent. However, the high volatility of SCFA (formate, acetate, propionate, and butyrate) impedes the use of a lyophilisation step and requires an NMR compatible solvent directly for extraction.

Sample stability at room temperature is another critical issue as: (a) single mice feces sample can weigh as little as 5 mg, which requires at least 30 min for NMR measurement at room temperature; (b) samples are measured in an automated mode using a sample changer, which often is not cooled. Thus our goal is to achieve sample stability at room temperature of minimum 6 hours and if possible 24 h to acquire the necessary quality of NMR spectra and efficiently use the measurement time. In past projects on urine, serum or fish larvae samples [24,25,26] this was achieved unless there was a problem with enzymatic and/or bacterial activity. A summary of the performed test extractions is given in Table 1 and Table 2 and their outcome is described in detail in the Results section.

## 2. Experimental Design

Mice in our study are fed either on a standard mice diet or a special diet (HFD) leading to increased lipid content in the corresponding feces. To minimize sample variation, a homogenization step mixing feces from different animals belonging to either the standard diet or the HFD group is applied at the beginning to generate identical test samples (pooled samples) used as aliquots throughout the manuscript. As the high fat diet leads to an increased amount of lipids in the mice feces, their signals can potentially mask the signals of the SCFA and so special care has been applied to take this into account. To generate test sample, feces have been thoroughly mixed by crushing them in a mortar, aliquoted, and kept at −20 °C until the actual extraction.

To increase stability at room temperature over several hours, a two phase extraction protocol is developed. An organic phase (deuterated) is added to the phosphate buffer. The clear phase separation of the organic and aqueous phases allows us to measure both extraction layers and to identify in which layer the desired SCFA is contained. The addition of organic solvents, however, extracts significant amounts of lipids into the aqueous phase rendering SCFA signal integration problematic, especially for HFD mice. In order to solve this problem, we have tried lipid background removal according to Kraus et al. with the crucial difference that we are interested in the aqueous and not the lipid containing organic phase [27]. 

**Figure 2:**
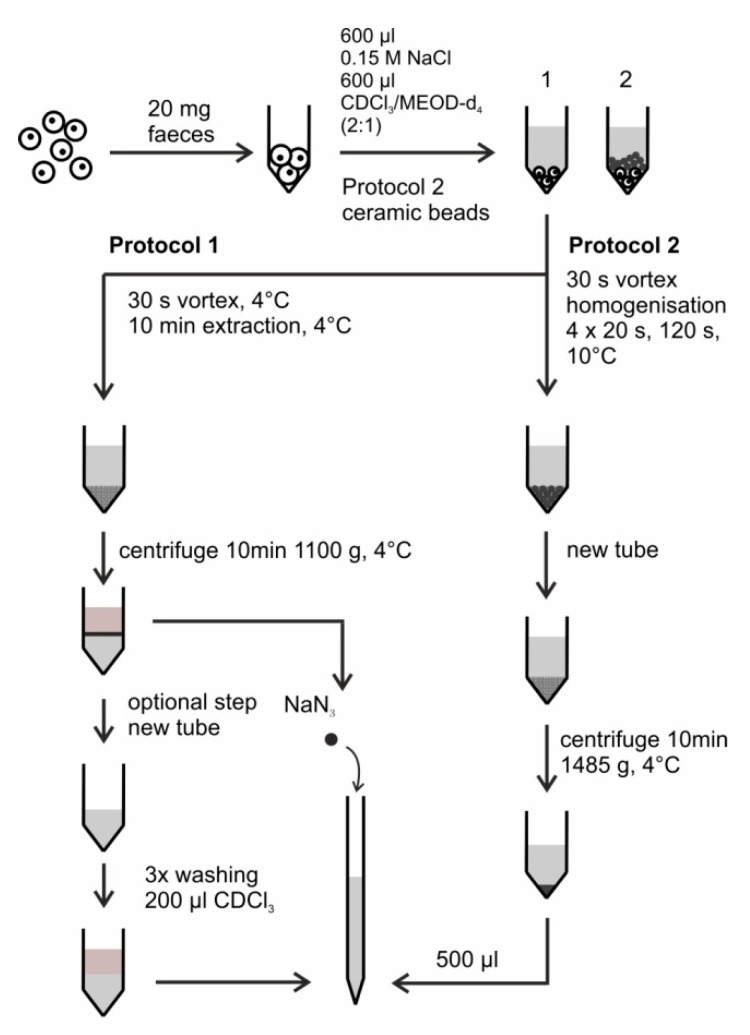
Schematic representation of the developed protocols for extraction of metabolites from mice feces within this work (Protocol 1 and Protocol 2). Protocol 1 has optional washing steps for more efficient lipid background removal. The difference of Protocol 2 with respect to Protocol 1 is the use of homogenizer.

As described in detail in the Results section, two versions of improved lipid extraction (LE) procedures with and without washing steps (Protocol 1) and the lipid extraction with homogenizer (Protocol 2) are developed. The two protocols perform equivalently in terms of removal of background signals and sample stability and which one is to be taken depends on the availability of a homogenizer. They are schematically demonstrated in Figure 2 and step by step explained in the Procedure section.

### 2.1. Materials

The mice feces originate exclusively from male C57Bl/6 mice purchased from Charles River (Sulzfeld, Germany) at 8 weeks of age. All experimental procedures are approved by the according authorities (regional council Karlsruhe regional council #35-9185.81/G-187/15) and carried out in the Central Animal Facility of the Max Rubner-Institut Karlsruhe in accordance with the German animal protection law. After one week of adaptation on a standard rodent chow, mice are fed on one of six experimental HFD, supplemented with 10% of different grain fractions or cellulose. The biological study investigates the influence of grain fractions on the development of nutrition based metabolic diseases and will be published elsewhere. Therefore two different kinds of feces are used for the development of this extraction protocol either from mice on standard rodent diet or on HFD. All the experimental diets have been specifically prepared for this study by Ssniff Spezialdiäten GmbH, Soest, Germany. All mice feces used within this manuscript have been randomly removed from the cages in the morning, frozen directly at −20 °C, stored for period between 4 and 8 months and homogenized before extraction by mortars as described in detail in the Procedure section. Thus these feces cannot be assigned to a specific animal number. 

In the extraction procedure with filters Amicon Ultra Centrifugal Filters with 3 kDa and 10 kDa are used from Merck KGaA, Darmstadt, Germany. 

The deuterated solvents are all obtained from Eurisotop GmbH. The deuterated water D_2_O has isotopic purity >99.90%, solvent MeOD-d_4_ and CDCl_3_ and acetonitrile-d_3_ are with isotopic purity >99.80%. For the buffer chemicals from Sigma-Aldrich with purity >99% are used. 1.5 M phosphate buffer is prepared from NaH_2_PO_4_/Na_2_HPO_4_, 20:80 D_2_O/H_2_O, 2mM TSP with pH adjusted to 7.40. For the saline solution 0.15 mM NaCl, 20:80 D_2_O/H_2_O, 2mM TSP are used and pH adjusted to 7.40. TSP is commonly used as a reference signal in NMR spectroscopy.

The chemicals used for extraction are as follows: NaH_2_PO_4_ (Sigma-Aldrich, Taufkirchen, Germany; Cat. no.: 71507-MM); Na_2_HPO_4_ (Sigma-Aldrich, Taufkirchen, Germany; Cat. no.: 71645-1KG); D_2_O (Eurisotop, Saarbrücken, Germany; Cat. no.: D214L); H_2_O: Millipore-Q biocel Adavantage A10 Merck KGaA Darmstadt, Germany; CDCl_3_ (Eurisotop, Saarbrücken, Germany; Cat. no.: D006H); TSP (Sigma-Aldrich, Taufkirchen, Germany; Cat. no.: 269913-1G); Acetonitrile-d_3_ (Eurisotop, Saarbrücken, Germany; Cat. no.: D021FE); MeOD-d_4_ (Eurisotop, Saarbrücken, Germany; Cat. no.: D021FE); EDTA (Sigma-Aldrich, Taufkirchen, Germany; Cat. no.: 798681-100G); NaN_3_ (Sigma-Aldrich, Taufkirchen, Germany; Cat. no.: S2002-25G); trichloroacetic acid (Sigma-Aldrich, Taufkirchen, Germany; Cat. no.: T6399-100G).

### 2.2. Equipment

Centrifuges: Eppendorf Centrifuge 5418 (Eppendorf AG, Hamburg, Germany), and Perfect Spin 24R (PEQLAB Biotechnologie GmbH, Erlangen, Germany)

Vortex: Mixer Vortex Genie 2 (Scientific Industries Inc., Bohemia, NY, USA) and Rotator with vortex RM – 2M (neoLab Migge GmbH, Heidelberg, Germany)

Ultra-sonic bath: FB 15047 (Fisher Scientific International Inc., Pittsburgh, PA, USA)

Homogenizer: Precellys Evolution Homogenizer (Berting GmbH, Frankfurt (Main), Germany) with Cryolys Cooling Option (Berting GmbH, Frankfurt (Main), Germany) and Precellys Lysing KITS (Berting GmbH, Frankfurt (Main), Germany; Cat. no.: CK 14).

NMR measurements: The NMR experiments are performed with a Bruker Avance II+ 600 MHz spectrometer (Bruker Biospin GmbH, Rheinstetten, Germany) equipped with a 5 mm room temperature BBI probe head with actively shielded z-gradients. The proton frequency of the used spectrometer is set to 600.193 MHz. The temperature stability is controlled via a Bruker BVT unit and is calibrated to be exactly 300 K. One dimensional proton experiments with water suppression are measured with the Bruker standard pulse sequence “noesygppr1D” for 1D NOESY experiment with water suppression and mixing time of 10 ms. The parameters used are as follows: 256 repetitions, four dummy scans, and a receiver gain set to 90.5. In some cases, additional experiments with Bruker standard pulse sequences “cpmgpr1D” and “jresgpprqf” are performed using standard parameters.

## 3. Procedure

### Time for Completion: Typically the Time for Completion Including Buffer Preparation Is 3:00 Hours.

Both protocols aim at an efficient and reproducible extraction of SCFA and other untargeted metabolites for NMR metabolomics studies, where long term (at least 24 h) stability at room temperature was targeted (in our case 20 °C). Both protocols use 20 mg homogenized mice feces. The fecal samples are frozen immediately after collection and stored at −20 °C until the extraction. The 0.15 M sodium chloride solution as described in the Materials section contains 20% D_2_O for the NMR lock system and 2mM TSP as a standard reference signal. The pH of the feces extracts is basic and depending on the origin (standard mice diet or HFD) varies between 7.9 and 8.1 pH.

**Protocol 1: Lipid Extraction (LE) Using Optional Washing Steps with Chloroform**

(1) Weigh 20 mg of the homogenized mouse feces into micro centrifuge tube. Unless otherwise specified and as far as possible, during all the waiting periods keep the samples on dry ice.
(2) Add 600 µL of 0.15 M sodium chloride to the tube. Add additional 600 µL of CDCl_3_/MeOD-d_4_ (2:1, v/v). Then vortex the mixture for 30 s.
(3) Place the micro centrifuge tube at 4 °C for the extraction period for 10 min.
(4) Centrifuge the suspension for 10 min at 1100× *g* and 4 °C.
(5) Transfer the aqueous phase to a new micro centrifuge tube for the washing procedure.

**Critical Step:** A critical step in this protocol is the separation of the two layers and pipetting of the aqueous phase (upper layer) from the organic phase (lower layer). The phase separation between the layers is very thin and contains residues of the mouse droppings. Thus it is easy to pierce into the organic phase and pipette together with the aqueous phase so that some of the organic layer could be transferred to the NMR tube.

(6) Add 200 μL of CDCl_3_ to the aqueous phase.
(7) Centrifuge the suspension for 2 min at 1100× *g* at 4 °C.
(8) Take the chloroform layer away and add fresh amount of 200 µL of CDCl_3_ to the aqueous phase. Repeat the centrifugation step 7.
(9) Take the chloroform layer again away and add fresh amount of 200 µL of CDCl_3_ to the aqueous phase.
(10) Perform the third centrifugation for 10 min at 1100× *g* at 4 °C.
(11) Take the chloroform layer again away. **Optional Steps:** The described steps from number 5 till 10 are optional. We have realized that these steps help removing the lipid content in our samples originating from mice fed on HFD. However, these steps could only be recommended when no additional small molecular sized metabolites could be also washed out in the process. This should be checked on the samples of interest. Alternatively, the number of washing steps could be reduced to either merely one or two instead of the tested three steps.
(12) Transfer 500 µL of the aqueous phase to an NMR tube, add NaN_3_ with 1.5 mmol/L concentration and measure the sample by NMR.

**Protocol 2: Lipid Extraction Using a Homogenizer**

(1) Weigh 20 mg of the homogenized mouse feces into the lysing kit tube CK 14 without beads.
(2) Add about 8 ceramic beads (one full spatula) to the solid feces material.
(3) Add 600 µL of 0.15 M sodium chloride to the tube. Additionally add 600 µL of CDCl_3_/MeOD-d_4_ (2:1, v/v).
(4) Vortex the suspension for 30 s.
(5) Seal the tube and homogenize it with a Precellys homogenizer. Perform four homogenization cycles at 6000 rpm at 10 °C. Each cycle lasts 20 s and is followed by a waiting time of 120 s at 10 °C.

**Critical Step:** A critical step in this protocol is the separation of the two layers and pipetting of the aqueous phase (upper layer) from the organic phase (lower layer). The phase separation between the layers is very thin and contains residues of the mouse droppings. Thus it is easy to pierce into the organic phase and pipette together with the aqueous phase so that some of the organic layer could be transferred in the NMR tube.

(6) Transfer the suspension to a new micro centrifuge tube and centrifuge for 10 min at 1485× *g* and 0 °C.
(7) Transfer 500 µL of the aqueous phase to a NMR tube, mix it with NaN_3_ with 1.5 mmol/L concentration and measure it by NMR.

**Pause Step:** Protocol 1 and 2 do not allow pause step within the extraction procedure. A possible pause could be performed after the aqueous phase has been transferred to the NMR tube and sodium azide is added. Then the NMR tubes could be stored on dry ice.

## 4. Results

Following the various phosphate buffer based extraction procedures, summarized in Table 1 and Table 2 [13,14], we have observed changes over time in some metabolites. In the Results section we describe the tested procedures, including the application of the protocol set developed within this manuscript and their outcome. Based on the fact that the two phase extraction protocol efficiently suppresses the changes over time, detectable by NMR spectroscopy, the changes are attributed to enzymatic activity. 

**Figure 3:**
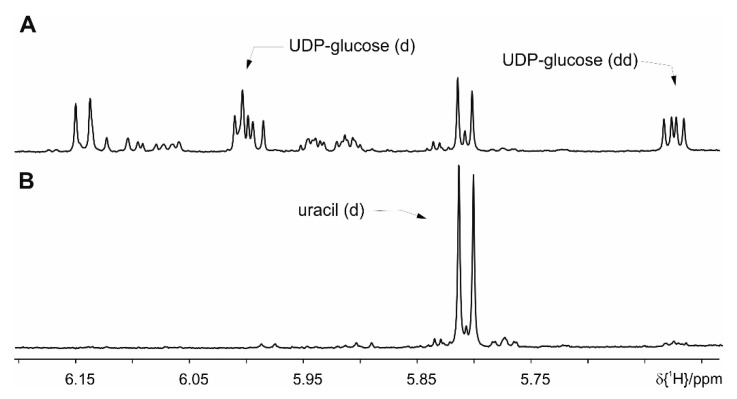
Comparison of the proton spectra taken within 24 h interval at room temperature: (**A**) fresh sample, (**B**) after 24 h.

The basic extraction protocol from the literature ([13,14] and references therein) comprises addition of buffer (700 µL) to the homogenized feces (20 mg), short vortexing followed by a centrifugation step, and transfer of the supernatant to a 5 mm NMR tube. The basic protocol is extended with various ultra-sonication steps [14], and/or freeze-thaw cycles with liquid nitrogen (shock freeze) [14], double extraction on the same feces amounts [13], and the addition of beads as beads allow efficient homogenization. A comparison of these extraction methods analyzed by NMR spectra is shown in Figures S1 and S2. Sample stability problems using these protocols have manifested themselves in an increase over the time of several amino acids’ peak intensities. As demonstrated in Figure 1, changes are observed for e.g., alanine (1.48 ppm, doublet (d)), threonine (1.33 ppm, d), valine (0.99 ppm, d, 1.05 ppm, d), leucine (0.96 ppm, d, 0.97 ppm, d), and isoleucine (0.94 ppm, t, 1.01 ppm, d). In addition to the increased amino acids, a decrease of UDP-glucose (5.62 ppm, dd, 6.00 ppm, d) can be observed. These changes are already detected in an immediate repetition of the measurement. Thus stability is less than 70 min, because 35 min are the minimum NMR measurement time per sample and we cannot even guarantee stability within the first measurement. After 24 h the two signals for the UDP-glucose and other signals between 5.50 and 6.20 ppm have disappeared completely (Figure 3). The latter changes most probably stem from bacterial activity as this problem could be solved by the addition of sodium azide (1.5 mmol/L). All following preparations always contain sodium azide. The original changes in amino acids are attributed to enzymatic activity (Figure 1). 

To identify and suppress enzymatic activity, we have tested different strategies to denature active proteins: (a) addition of a range of ethylenediaminetetraacetic acid (EDTA) concentrations up to a final concentration of 2 mM to inhibit metalloproteases; (b) heating briefly to 40 °C, 60 °C, or 90 °C for 10 min; addition of (c) acetonitrile at three different concentrations or (d) of trichloroacetic acid (TCA) solution (20 µL) to the extraction phosphate buffer (680 µL) to precipitate proteins. Other protease inhibitors apart from EDTA have not been tried because their signals would severely overlap with several metabolites.

**Figure 4:**
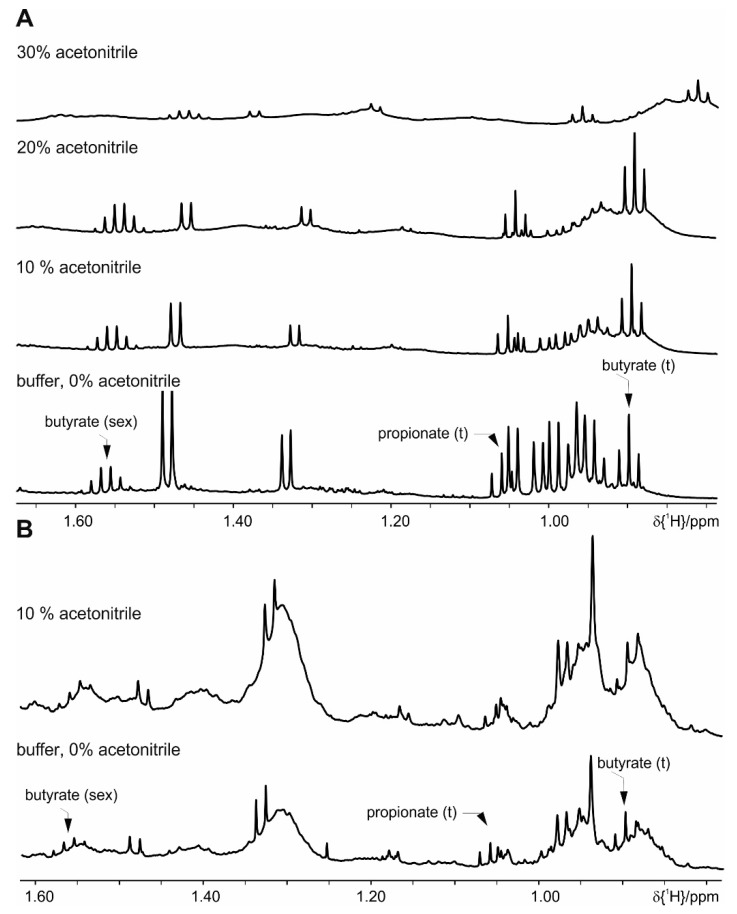
Effect of extraction with different concentrations of acetonitrile on the metabolite signals: (**A**) feces of mice fed on a standard diet and (**B**) on a HFD diet. For clarity only a selected spectral range of the proton NMR spectra is shown. Please note that the increased amount of acetonitrile induces chemical shifts on the signal resonances.

The addition of 20% deuterated acetonitrile slows down but does not fully prevent enzymatic activity over 24 h. Addition of 30% (or higher amounts of) acetonitrile causes measurement problems due to the presence of a second deuterated solvent, which prevents automatic measurement. As additional drawback, signals show severe line broadening and accurate integration is not possible (Figure 4). Protein denaturation by pH change, addition of TCA or EDTA is not successful to suppress enzymatic activity. Heating to 60 °C for 10 min results in both an increase of the background signals (Figure S3) and still shows residual enzymatic activity after 5 h. Heating to 90 °C for 10 min shows sample stability for more than a day, but increased strong background signals of protein and/or lipids prevents proper integration and risks loss of the volatile SCFA.

**Figure 5:**
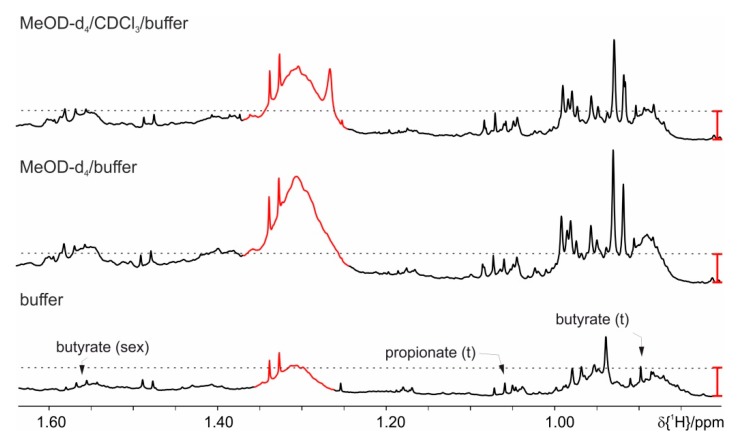
Comparison of different extraction procedures: basic protocol (buffer) and the two-phase extraction protocols (with and without chloroform). The red bar indicates the signal intensity of the extracted background signal at 1.31 ppm and is kept the same for the two-phase protocols.

The two phase protocol with a mixed MeOD-d_4_/CDCl_3_/phosphate buffer according to [14] extracts SCFA metabolites in the aqueous phase. This shows no bacterial and enzymatic activity over time (up to 24 h). The exchange of chloroform to dichloromethane leads to similar results, whereas omission of chloroform during extraction leads to an increase in lipid background (Figure 5). As depicted in Figure 5, the SCFA signals have lower intensity on an increased lipid background. This overlap causes integration problems. When comparing the intensity of the extracted butyrate with the basic protocol and the two phase extraction, it becomes clear that butyrate is so highly overlapped that it becomes undetectable with the two phase extraction protocol. In addition, the presence of more than one deuterated solvent (CDCl_3_, MeOH-d_4_, and D_2_O) leads to random lock switches in the automatic measurements and consequently to severe problems in water suppression and arbitrary settings of the chemical shift scale. An additional drawback of this protocol is the long preparation period of about 24 h in total, including storage step in a refrigerator overnight (approximately 16 h), which makes it impractical for studies with large number of samples. An extraction with cold methanol only is completely inefficient (Figure S4).

**Figure 6:**
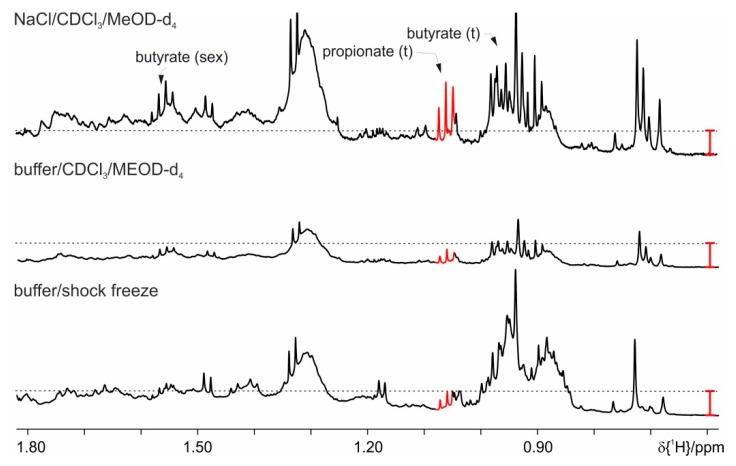
Proton spectra for the two phase protocols, on feces from mice on HFD, where basic protocol with shock freeze (buffer/shock freeze) is compared with a lipid extraction performed with buffer and organic phase or with the use of sodium chloride. The red bar indicates the increased signal intensities.

**Figure 7:**
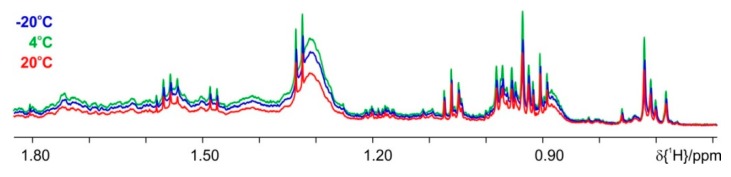
Influence of extraction temperature. Comparison of proton spectra, where the extraction performed at −20 °C is given in blue, the 4 °C extraction in green and the one at +20 °C in red color.

To overcome the problem with increased lipid background and eventually reduced SCFA extraction, we have looked for procedures to shift the lipid extraction completely to the organic phase. A suitable protocol is found in Kraus et al. [27]. A comparison of the NMR spectra from this protocol (sodium chloride and a mixed solvent of chloroform and methanol) with phosphate buffer extraction shows both increased amounts of SCFA and the desired stability of the samples over time (Figure 6). The drawback of this protocol is again increased background, originating from lipids and probably steroids, in the aqueous phase, thus for further optimization the influence of temperature is evaluated. Extraction and the centrifugation step after vortexing are tested at either −20 °C, 4 °C, or +20 °C (Figure 7). In order to achieve better and clearer phase separation increased centrifugation speed up to 11,000× *g* is also evaluated, but has no positive influence. The optimum result is obtained when extraction and centrifugation steps are performed at 4 °C (see Figure 7). Increased amounts of the SCFA could be obtained in this way with concomitant reduction in lipid background. Protocol 1 (a) shows the suppression of the enzymatic activity; (b) allows extraction of maximum amounts of metabolites; (c) the contribution of background signals is kept to minimum and (d) it performs equivalently well for feces with different fat composition, e.g., mice on standard diet and HFD. 

As the application of this protocol allows fulfilling all the requirements, we have evaluated its performance by comparing the NMR spectra of the two phases. After phase separation, the chloroform phase is measured and plotted against the aqueous phase (Figure S5). It confirms our expectation that lipids would predominantly reside in this phase. 

As residual lipid background could be identified in the spectra after the optimization of Protocol 1, we have tried further washing steps with chloroform to remove more of the lipid content from the aqueous phase. Each washing step is followed by centrifugation, where the supernatant is taken for further washing. The final centrifugation is longer to allow better phase separation. At the end the aqueous phase is transferred to a NMR tube for measurements. This we refer to Protocol 1 with optional washing steps.

**Figure 8:**
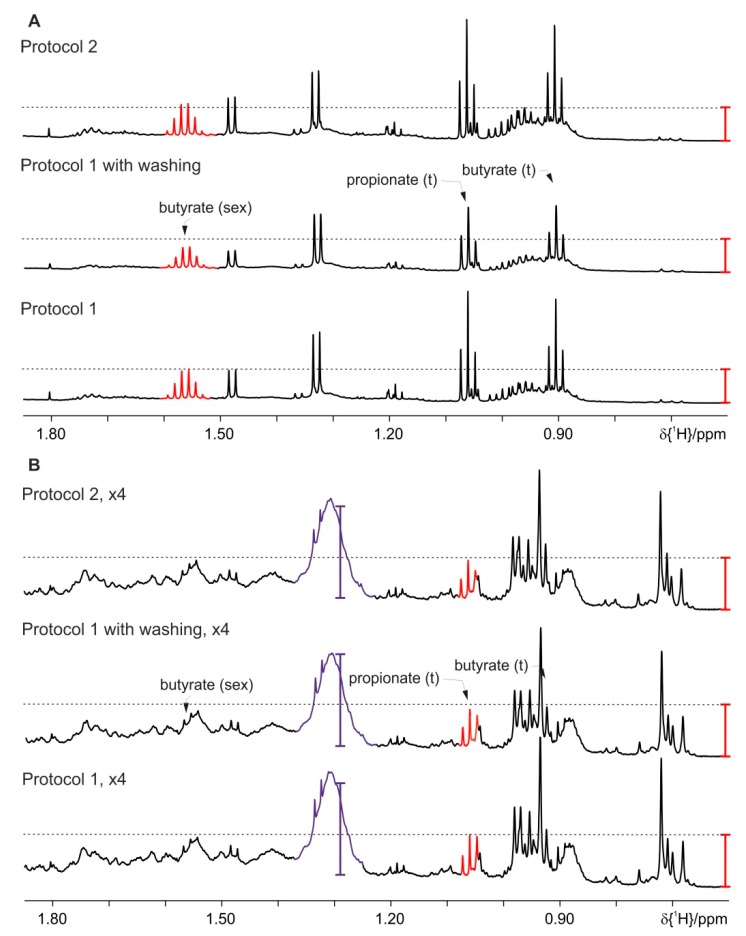
Effect of optimized lipid extraction at 4 °C on the concentration of metabolites and amounts of background signals using Protocol 1 without and with washing steps and Protocol 2: (**A**) feces of mice fed on a standard diet and (**B**) on HFD (the intensity is upscaled four times). For clarity only a selected spectral range of the proton NMR spectra is shown. The red and violet bars are fixed at the same height in each section of the figure and indicate the increased signal intensities of the SCFA and background signals, respectively.

The washing steps remove the reminder of background signals even for HFD mice (Figure 8, section B) but as a clear drawback of other metabolites, mostly amino acids and sugars, also decrease. Figure 8, section A (mice on standard diet) shows that the signals for the SCFA have decreased intensity after the washing steps. Thus further optimization using a homogenizer for extraction is tried (Protocol 2). The feces together with the solvents are placed into Precellys tubes with ceramic beads. After vortexing the sample is homogenized in accordance to the homogenizer’s manufacturer instructions. The mixed solution is transferred to a micro centrifuge tube, centrifuged and the aqueous upper phase is filled into a 5 mm NMR tube for measurement. The resulting spectra for the homogenized mice feces on standard diet and HFD are given in Figure 8. It is worth noting that the background signal (indicated in purple) interferes with the integration of metabolites in this area, typically amino acid signals.

**Figure 9:**
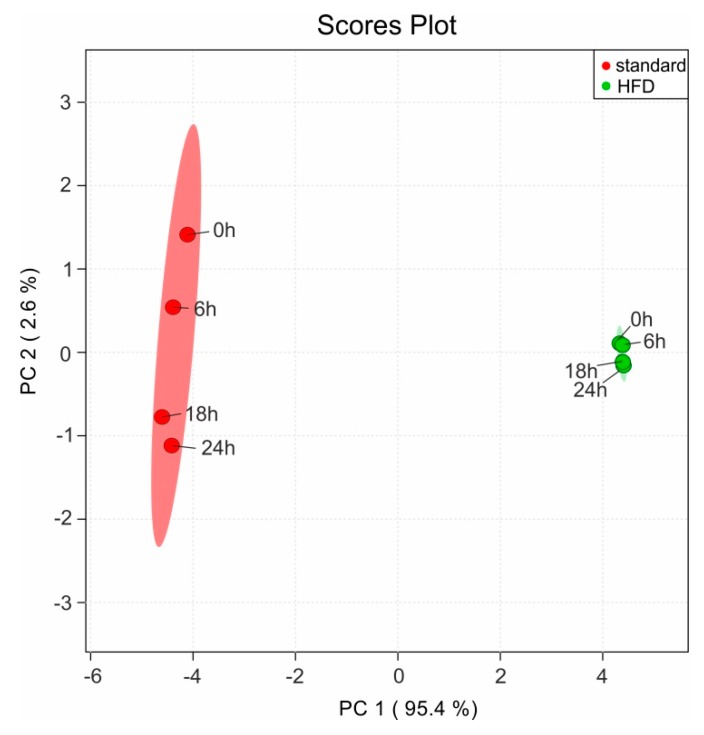
Statistical analysis of the variations in the NMR spectra over time using a PCA. The scores plot represents the explained variations in the samples for mice fed on standard diet (in red) and on HFD (in green) recorded at four different time points (0 h, 6 h, 18 h, and 24 h) with storage at RT.

We have evaluated the samples stability at RT in the sample changer (set in our case to 20 °C) by recording a new NMR measurement every six hours (0 h, 6 h, 18 h, and 24 h) for both sample types: extracts with Protocol 2 from mice fed on standard and HFD diet. The NMR spectra are afterwards read into the software Amix 3.9.14, where a pattern file with the peaks from the complete NMR spectra is created. For these peaks the respective bucket table is generated in Amix. Thus in the bucket table each signal is represented as a separate bin and it can be compared with the same spectral pieces from the other NMR measurements. The bucket table is afterwards used for a statistical evaluation (principal component analysis, PCA) of the observed variations in the NMR spectra over time using the online MetaboAnalyst package [28,29,30,31]. Range scaling is applied to each bin prior to the statistical analysis, which ensures that all bins are treated equally [32,33]. The explained variation with the PC1 is 95.4% is as expected due to the differences based on the diet. The remaining 2.6% variation on the PC2, as it can be seen in the resulting PCA scores plot (Figure 9) demonstrates the minimal variation observed on the same sample over time at RT. To some extend these minor variations in the consecutive NMR measurements can be attributed to the performance of the automated field homogeneity routine, leading to line broadening in the observed resonances. This result clearly indicates that the extracted solution using the protocol set presented in this manuscript does not show significant changes (neither chemical degradation, nor enzymatic activity) in the NMR spectra when stored at room temperature up to 24 h.

**Figure 10:**
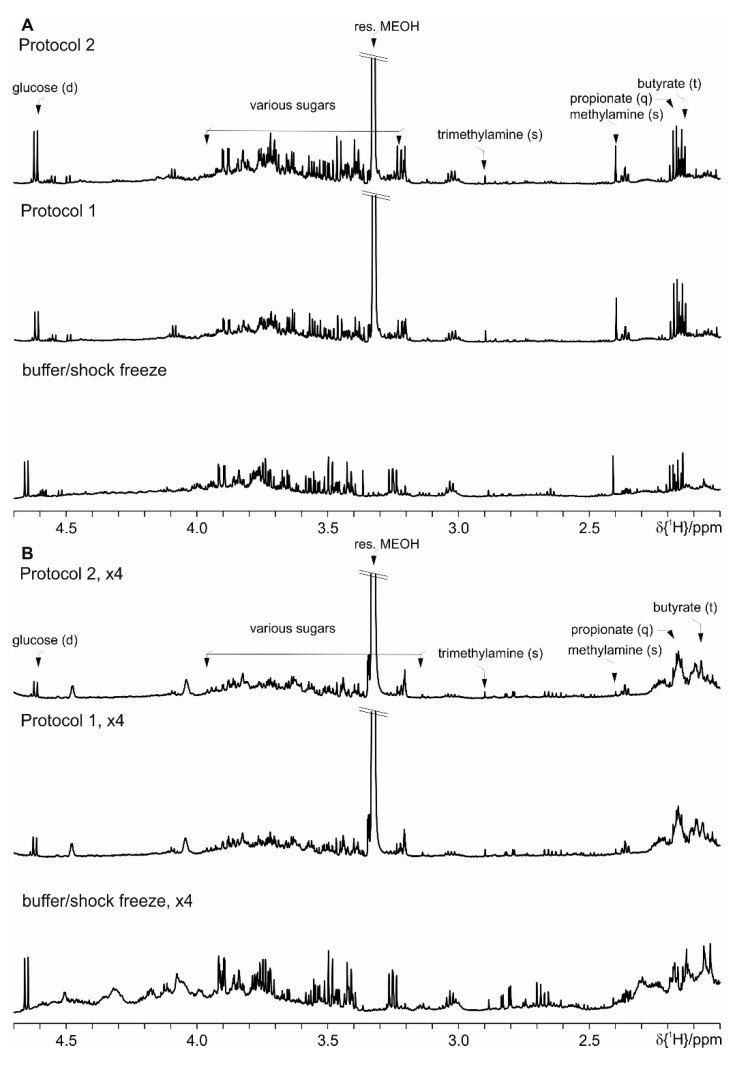
Comparison of Protocol 1 and 2 with the basic protocol with shock freeze: (**A**) feces of mice fed on a standard diet and (**B**) on HFD (the intensity is upscaled four times). For clarity only a selected spectral range (2.0 ppm till 4.7 ppm) of the proton NMR spectra is shown, where the most prominent metabolites are marked.

**Figure 11:**
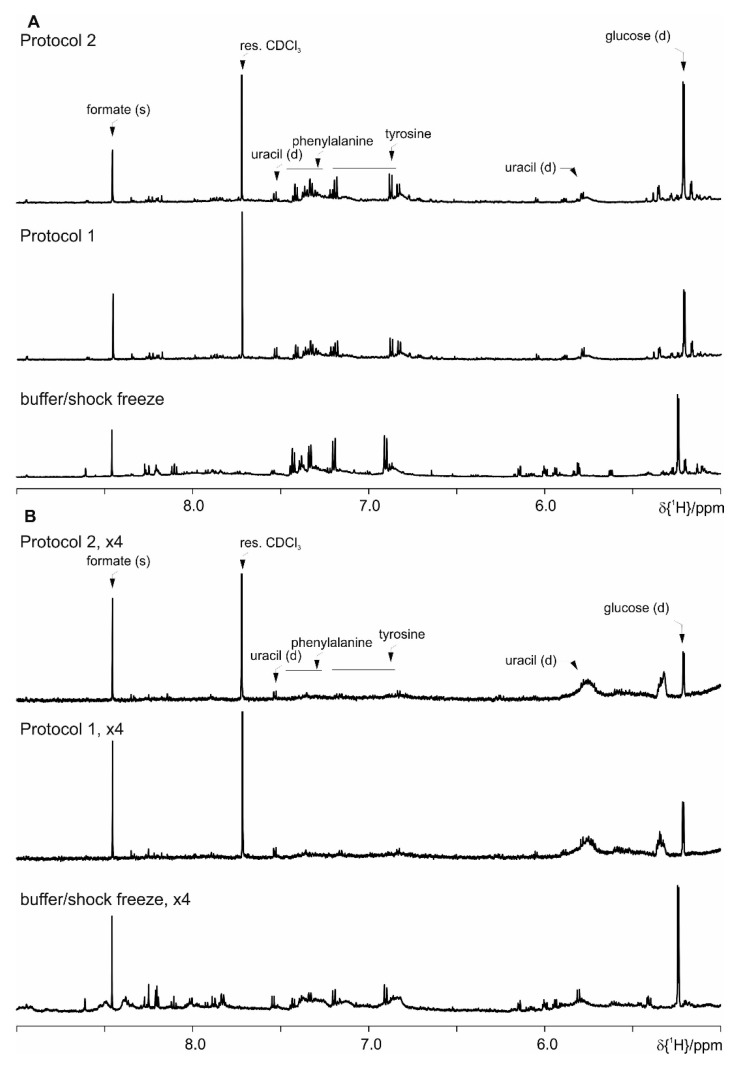
Comparison of Protocol 1 and 2 with the basic protocol with shock freeze: (**A**) feces of mice fed on a standard diet and (**B**) on HFD (the intensity is upscaled four times). For clarity only a selected spectral range (5.0 ppm till 9.0 ppm) of the proton NMR spectra is shown, where the most prominent metabolites are assigned.

Even though the focus for the development of this protocol set is the observation of the SCFA, we have further evaluated the performance of Protocol 1 and Protocol 2 versus the basic protocol with the shock freeze extraction. As it can be seen in the following figures (Figure S6 where spectral ranges from 0.6 till 2.0 ppm are shown, Figure 10—from 2.0 ppm till 4.7 ppm and Figure 11—from 5.0 ppm till 9.0 ppm), both here developed protocols allow the extraction of comparable number of metabolites. Merely the most prominent metabolites are indicated as the complete spectral assignment will be published together with the results of the biological study elsewhere. Thus these protocols are as well suitable for untargeted NMR metabolomics studies. 

**Figure 12:**
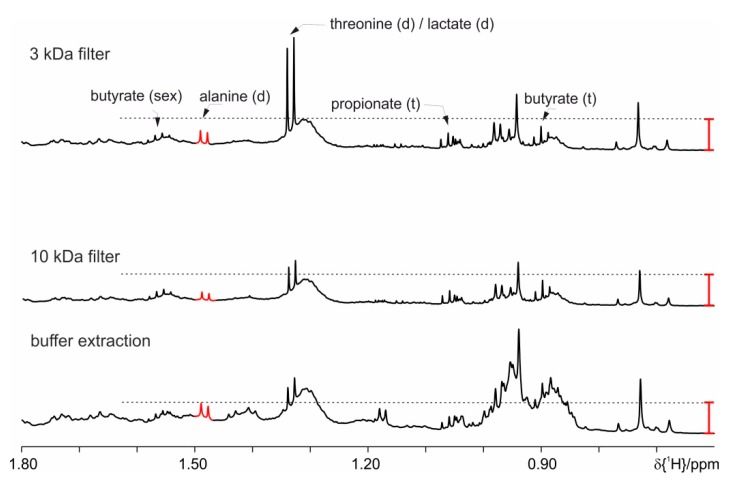
Proton spectra of feces extraction from mice fed on study diet (HFD) demonstrating the effect of the use of cut off filters (3 kDa and 10 kDa filter) to separate the high molecular weight components. As comparison, a spectrum obtained with the basic protocol is shown. The red bar indicates the differences in the signal intensities.

As a final option, we have used filters with a suitable cut off molecular size to separate the signals from high molecular weight lipid and proteins compounds (3 kDa and 10 kDa). The 3 kDa filter shows increased intensity for the signal at 1.33 ppm (Figure 12), either lactate or threonine. This could be due to the increased time because of the centrifugation step during which the proteases can be active. Samples prepared with a filter centrifugation step have no changes after 20 h, while 10 kDa filters allow more than 4 days stability. With this approach, the best performance is achieved compared to all previously tested approaches. Nevertheless, one drawback of this filtering approach concerns butyrate (0.82 ppm, t) as there is remaining background signal (Figure 12). The thereby introduced integration error is non-systematic and will lead to improper statistical evaluation of the NMR spectra. We have restrained ourselves from using the filters as they cannot be reused, are relatively expensive and thus are not the first choice for studies with more than 300 samples.

We have adapted Protocol 1 for those labs where a homogenizer is available and referred to it as Protocol 2. As it can be seen in Figure 5 and in Figure S6, Figure 10 and Figure 11, Protocol 1 without the optional washing steps and Protocol 2 (with homogenizer) lead to practically overlapping spectra for both mice feces types. The use of a homogenizer leads to less and simpler steps and extracts a maximum amount of SCFA with minimal background disturbance in an efficient and easy way.

As additional safety precaution to overcome the initial problem with bacterial activity, sample storage should avoid unnecessary periods at RT. Probably storage at −20 °C is not sufficient. An optimal procedure in our lab is immediate shock freeze by liquid nitrogen with following storage at −80 °C. The protocol versions presented in this manuscript (Protocol 1 and 2) with or without the optional washing steps allow sample storage after extraction for 24 h. They are cost efficient as they do not require the use of too expensive consumables. The two phase extraction allows the suppression of undesired enzymatic activity and they are suitable for measurements of the typical number of hundreds to thousands of samples in metabolomics studies. 

## Figures and Tables

**Table 1:** Summary of the single-phase buffer extraction protocols used for the homogenized mice feces.

Protocol	Freeze Cycle ^1^	Ultra-Sonication	Details	Reference
Basic			30 s vortex	Wu [13]
ultrasonic		3 × 30 s	kept at 4 °C, vortex in between	Lamichhane [14]
Shock freeze ^1^	3 × 30 s		vortex before freezing	Lamichhane [14]
Combination of ultra-sonication and shock freeze ^1^	3 × 30 s	3 × 30 s	kept to 4 °C vortex in between the runs	Lamichhane [14]
Double extraction, 1st fraction		3 × 30 s	kept at 4 °C, vortex in between	Wu [13]
Double extraction, 2nd fraction		3 × 30 s	kept at 4 °C, vortex in between	Wu [13]
Double extraction, fractions combined		3 × 30 s	kept at 4 °C, vortex in between, lyophilised at −80 °C overnight	Wu [13]
Beads			ceramic beads 15 min by 99 rpm juddered	
Acetonitrile (10%, 20%, or 30%)		3 × 30 s	tempered to 4 °C vortex in between	
EDTA			2 mM EDTA, 30 s vortex	
8.75 M TCA/buffer (1:34 v:v)			10 min incubation in fridge (6 °C)	
Heating (40 °C, 60 °C or 90 °C)			10 min	
Filter (10 kDa or 3 kDa)			Supernatant 15 or 30 min at 15,000× *g* centrifuged	

^1^ with liquid nitrogen.

**Table 2:** Summary of the two phase extraction protocols used for the homogenized mice feces.

Extraction	Solvent ^1^	Centrifugation	Details	Reference
MeOD-d_4_/CDCl_3_/D_2_O (or buffer ^2^)	300/300/350 (1:1:1.166)	30 min, 1400× *g*, 4 °C, 10 min 15,000× *g*, 4 °C, each separate phase	Phase separation overnight	Lamichhane [14]
CD_2_Cl_2_/buffer	600/600 (1:1)	30 min 15,000× *g*, 4 °C, aqueous phase 10 min 15,000× *g*, 4 °C	-	-
0.15 M NaCl/CDCl_3_/MeOD-d_4_	600/400/200 (3:2:1)	RT, 10 min, 1100× *g*	-	Kraus [27] ^3^
0.15 M NaCl/CDCl_3_/MeOD-d_4_	600/400/200 (3:2:1)	30 s vortex, 4 °C, 10 min, 1100× *g*	Optionally three CDCl_3_ washing steps	Protocol 1, this work
0.15 M NaCl/CDCl_3_/MeOD-d_4_	600/400/200 (3:2:1)	30 s vortex, homogenizer four times 20 s, 10 °C, 6000 rpm, centrifuged 0 °C 1485× *g* for 10 min	-	Protocol 2, this work

^1^ The amounts of solvent are given in µL and as ratios in brackets; ^2^ buffer means phosphate buffer; ^3^ for extraction of hydrophobic components using the chloroform phase.

## Supplementary Materials

The following are available online at https://www.mdpi.com/2218-1989/9/3/55/s1: Figure S1: Comparison spectra of the extracted metabolites using ultra-sonication and/or shock freeze for extraction of mice feces on a standard diet. For clarity only the spectral region of interest, harbouring resonances of the short chain fatty acid (SCFA) is shown, Figure S2: Comparison spectra of the extracted metabolites using single extraction steps and their combination, where first and second extraction is performed on mice feces on a standard diet and the combined phase of the extractions after lyophilisation is given. For clarity only the spectral region of interest, harbouring resonances of the SCFA is shown. Figure S3. Evaluation of the influence of the temperature on the recorded NMR spectra, where in green is the spectrum with the phosphate buffer extraction at room temperature, blue with heating to 40°C, 60°C (violet), and 90°C (red) colour. Figure S4. Comparison of the methanol extraction applied to feces from mice on standard diet, where the signal intensities in the spectrum are scaled down eight times, and from mice on high fat diet. Figure S5. Comparison of the metabolites in the aqueous and chloroform phases originating from optimised protocol for lipid extraction, referred to as Protocol 1. Figure S6. Comparison of Protocol 1 and 2 with the basic protocol with shock freeze: (A) feces of mice fed on a standard diet and (B) on high fat diet (HFD) (the intensity is upscaled four times). For clarity only a selected spectral range (0.6 ppm till 2.0 ppm) of the proton NMR spectra is shown and the most prominent metabolites are assigned. Protocol 1 and 2 clearly extract more efficiently the SCFA, based on their increased intensity for both diets. Figure S7. 1H NMR spectrum of the extracted metabolites from mice on a standard diet with phosphate buffer protocol with shock freeze. Figure S8. 1H NMR spectrum of the extracted metabolites from mice on a HFD with phosphate buffer protocol with shock freeze Figure S9. 1H NMR spectrum of the extracted metabolites from mice on a HFD with ultrafiltration (3kDa cut off filter). Figure S10. 1H NMR spectrum of the extracted metabolites from mice on a standard diet with Protocol 1 without washing steps. Figure S11. 1H NMR spectrum of the extracted metabolites from mice on a HFD with Protocol 1 without washing steps. Figure S12. 1H NMR spectrum of the extracted metabolites from mice on a standard diet with Protocol 2. Figure S13. 1H NMR spectrum of the extracted metabolites from mice on HFD with Protocol 2.
