# Supplementary material for: Efficient Extraction from Mice Feces for NMR Metabolomics Measurements with Special Emphasis on SCFAs
Source: Metabolites. 2019 Mar 21;9(3):55. doi: 10.3390/metabo9030055 (PMC6468719; doi:10.3390/metabo9030055)
Supplement: Supplementary file 1 [file metabolites-09-00055-s001.pdf]

Supplementary Materials

Efficient Extraction from Mice Feces for NMR Metabolomics Measurements with Special Emphasis on SCFAs

Adrian Hauser, Philipp Eisenmann, Claudia Muhle-Goll, Burkhard Luy, Andreas Dötsch, Daniela Graf and Pavleta Tzvetkova

The detailed explanation of the protocols we have tried is separated into two major sections: one phase, where phosphate buffer based protocols are given and a second section, where two phase or organic phase containing protocols, are explained in details.

- **Section 1:** One phase, buffer extraction based protocols:

1. Basic extraction protocol:

   Buffer extractions from the homogenized mice feces material in slightly different variation are performed. Typically, 20 mg homogenized mice feces material is balanced to which 700 µl phosphate buffer are added. Afterwards mixing for 30 s with vortex mixer is performed. The suspension is centrifuged at 4°C and 15 000 x g for 10 minutes. 500 µl of the supernatant are taken and transferred into 5 mm NMR tubes for measurement.
2. Variations of the basic protocol:

   The extension of the basic protocol includes the use of various ultra-sonication steps, and/or freeze-thaw cycles with liquid nitrogen referred to as shock freeze, double extraction on the same feces amounts, and beads addition in accordance to the summary of performed test extractions given in Table 1 in the main text. As we wanted to compare the influence of the extraction efficiency we have varied one parameter at the time. Thus the differences here to the basic protocol are either in the performance of shock freeze cycle addition, the addition of ultra-sonication steps or the combination of both, while keeping the vortexing and centrifugation steps exactly the same. The comparison of the extracted metabolites is given in Figure S1 below:

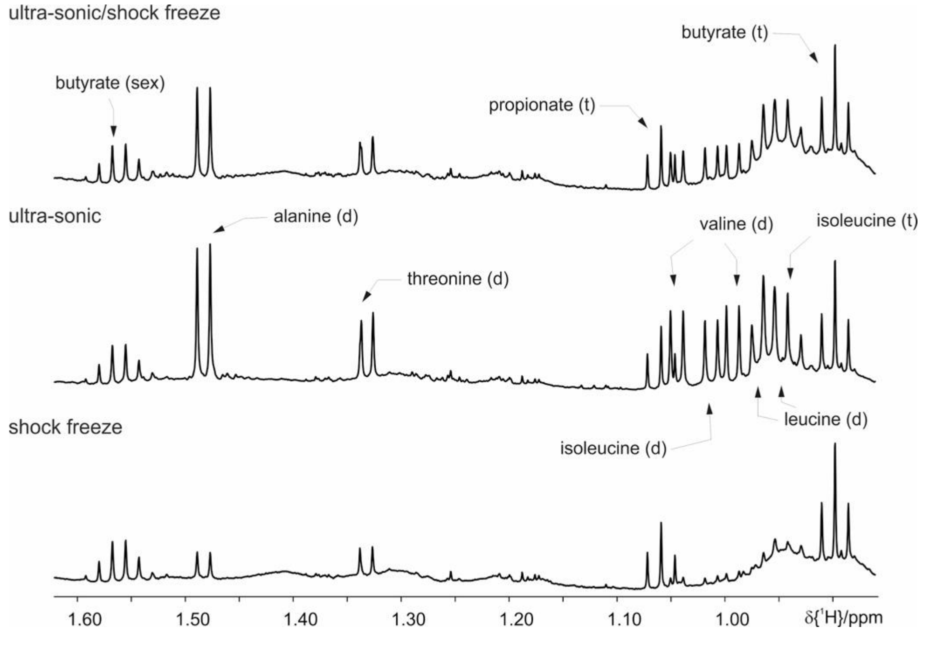

Figure S1. Comparison spectra of the extracted metabolites using ultra-sonication and/or shock freeze for extraction of mice feces on a standard diet. For clarity only the spectral region of interest, harbouring resonances of the short chain fatty acid (SCFA) is shown.

3. Combination of double extraction cycles

The experiments are performed in accordance to the protocol suggested by Wu et al. [1], where the extraction is repeated to the same feces material twice and the two extraction supernatants are combined and further lyophilised. In Figure S2 is shown the outcome from the first and second extraction and as well the NMR spectrum of the combined phase.

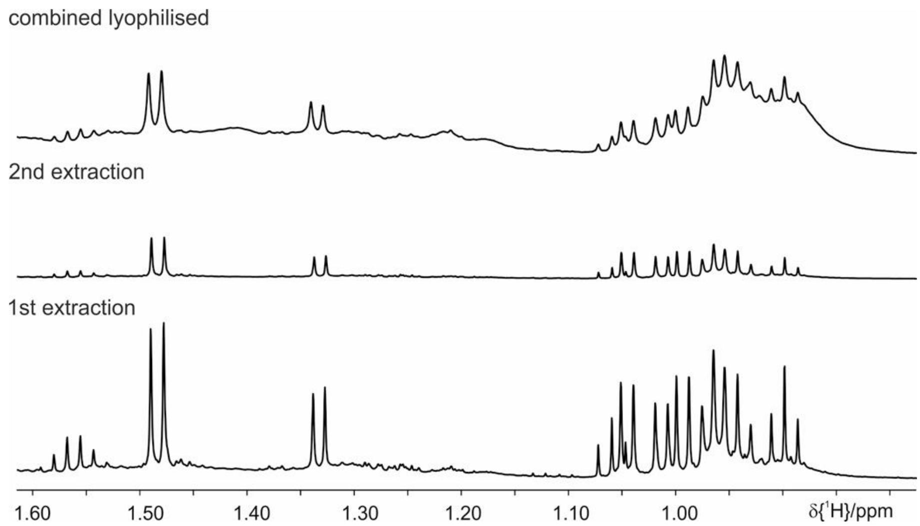

Figure S2. Comparison spectra of the extracted metabolites using single extraction steps and their combination, where first and second extraction is performed on mice feces on a standard diet and the combined phase of the extractions after lyophilisation is given. For clarity only the spectral region of interest, harbouring resonances of the SCFA is shown.

4. The phosphate buffer extraction is tested with the addition of EDTA, TCA, and acetonitrile and with heating up the sample. There are three different temperatures tested: 40°C, 60°C, and 90°C with the aim to prohibit the protease activity. An increase of the background signals from 60°C upwards (Figure S3) is observed.

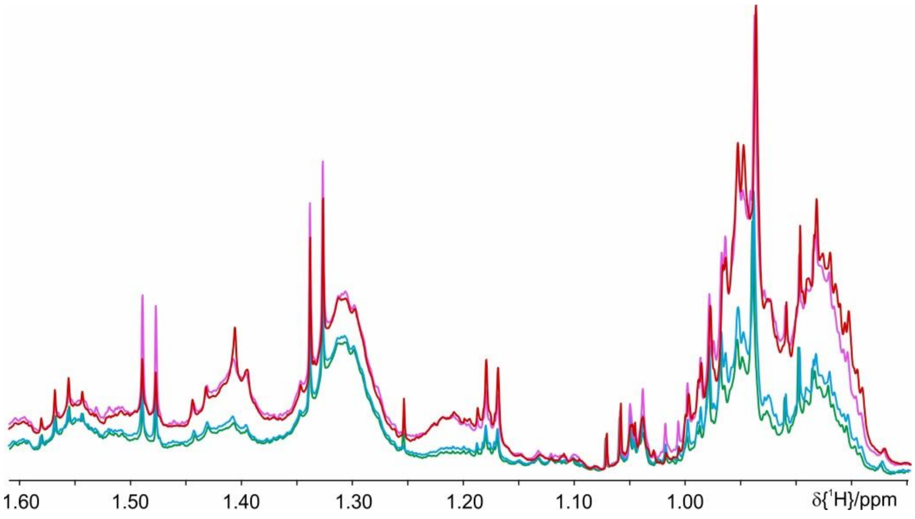

Figure S3. Evaluation of the influence of the temperature on the recorded NMR spectra, where in green is the spectrum with the phosphate buffer extraction at room temperature, blue with heating to 40°C, 60°C (violet), and 90°C (red) colour.

- **Section 2:** Protocols based on organic phase extraction in one or two phases:

1. Mixed organic solvent extraction:

The two-phase extraction protocol includes the use of mixed organic solvents as follows: to the 20 mg mice feces sample 300 µl cold methanol-d4 are added, where the sample is mixed with a vortex for 30 s and further cooled down in an ice bath for 10 minutes. Then 300 µl $CDCl_3$ and 350 µl D_2O or phosphate buffer are respectively added. After a new 30 s vortexing the solution is left overnight in the fridge. The next day the solution is centrifuged for 30 minutes at 4 °C and 1400 x g [2] and reference therein. Both the aqueous and organic phases are transferred in micro centrifuge tubes and centrifuged (at 4 °C for 10 min at 15000 x g). The chloroform phase is up scaled to 500 µl to fully fill the 5 mm NMR tubes. The target metabolites are identified in the aqueous phase and in order to understand the chloroform influence the same protocol steps are repeated omitting the addition of chloroform. The corresponding NMR experiments are shown in Figure 5 in main text.

2. Methanol extraction

In an attempt to simplify the multi solvent extraction a simple methanol extraction is tested, where cold methanol-d4 is added the mice feces, followed by vortexing, an ice bath extraction time and centrifugation. 700 µl of cold methanol-d4 are added to 20 mg mice feces. After a 30 s mixing with the vortex, the sample is placed in an ice bath for 10 minutes and then centrifuged at 4°C and 15000 x g for 10 minutes. The supernatant is taken and transferred to a 5 mm NMR tube. 2 mM TSP is added as a reference signal. This simple protocol has allowed us to stop bacteria and the enzymatic activity, however, the extraction is quite inefficient in the case of mice feces on high fat diet as shown in Figure S4. In the case of HFD mice samples the amounts of extracted SCFA are at the level of noise and the comparison with the spectrum of mice on standard diet requires at least eight times downscaling (Figure S4). Yet, a high level of lipid background is extracted in both types of samples. In addition, the NMR spectrometer has problems with correctly locking on the desired solvent peak.

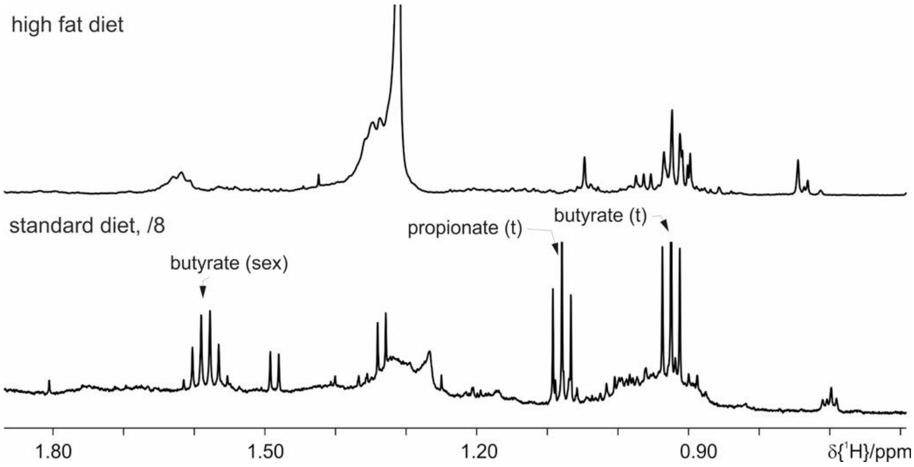

Figure S4. Comparison of the methanol extraction applied to feces from mice on standard diet, where the signal intensities in the spectrum are scaled down eight times, and from mice on high fat diet.

3. Lipid extraction protocols

A mice feces lipid extraction is performed according by Kraus *et al.* using 600 µl 0.15 M sodium chloride solution is mixed with the mixture of chloroform-d and methanol-d4 (2:1) at RT [3]. This protocol was applied as described by Kraus *et al.* and since the mixed solvents prevented the sample changes over time, it serves as a basis and was further optimized. Here, we describe below in details all the optimisation steps. We have tested the lipid extraction by using either phosphate buffer or sodium chloride solution, performing the extraction steps at various temperatures and the centrifugation conditions. The exact procedure is the following: to the mice feces are added 600 µl 0.15 M sodium chloride (or phosphate buffer) and 600 µl of a 2:1 volume mixture of $CDCl_3$ and methanol-d_4. Optimal extraction is tested on identical sample sets kept at either -20 °C, 4 °C or +20 °C. The samples are vortexed for 30 s. As we are targeting at the SCFA, we have tested the temperature for centrifugation to -20 °C, 4 °C or, +20 °C for one sample per set. Thus we have had tested all variations for cold or RT extraction followed by either cold or RT centrifugation. In order to achieve better and clearer phase separation an increased centrifugation speed is evaluated. It is not found beneficial to increase the centrifugation to 11000 x g. The comparison of the optimal temperature for the extraction showed that 4 °C is most optimal as maximal amount of extracted SCFA versus minimal background signals could be achieved (see Figure 7, main text). The optimal result of the above optimisation and what we called Protocol 1 for lipid extraction was achieved with extraction at 4 °C and centrifugation at the same temperature.

As the amounts of background signals are still high, in addition we have performed further two washing steps with chloroform to remove the lipid content from the aqueous phase. After each washing step with the addition of 200 µl $CDCl_3$, the samples are centrifuged at 4°C and 1100 x g for 2 minutes. At the end additional 200 µl $CDCl_3$ are added and the samples are centrifuged at 4°C and 1100 x g for 10 minutes. Together with the addition of a crystal NaN_3 500 µl of the aqueous phase are transferred to a NMR tube for measurements. This procedure we refer to as Protocol 1 with washing steps. As a comparison we have performed equivalent phosphate buffer extraction instead of 0.15 M sodium chloride in a second iteration for the protocol optimization and we have identified the sodium chloride version as better suited for extraction of SCFA from mice feces as shown in Figure 6 in main text.

In final optimisation iteration we weight the 20 mg of feces into Precellys tubes with ceramic beads. The 600 µl sodium chloride (0.15 M) and 600 µl mixture with $CDCl_3$ and MeOD-d_4 (2:1, $CDCl_3$:MeOD-d_4) are added into the tube. The mixture is vortexed for 30 s. As a next step the sample is homogenised four times for 20 s at 10°C with 6000 rpm with a waiting time of 120 s in the homogenizer. The mixed solution is transferred in a micro centrifuge tube and centrifuged at 0°C and 1485 x g for 10 minutes. 500 µl of the aqueous upper phase are taken and filled in 5 mm NMR tube for measurement with the addition of a crystal NaN_3. This procedure we refer to as Protocol 2.

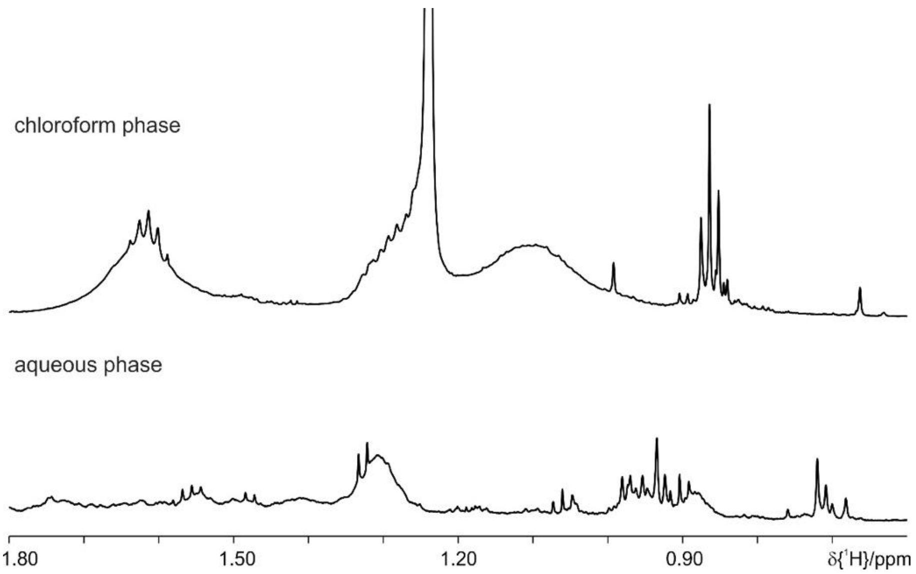

Figure S5. Comparison of the metabolites in the aqueous and chloroform phases originating from optimised protocol for lipid extraction, referred to as Protocol 1.

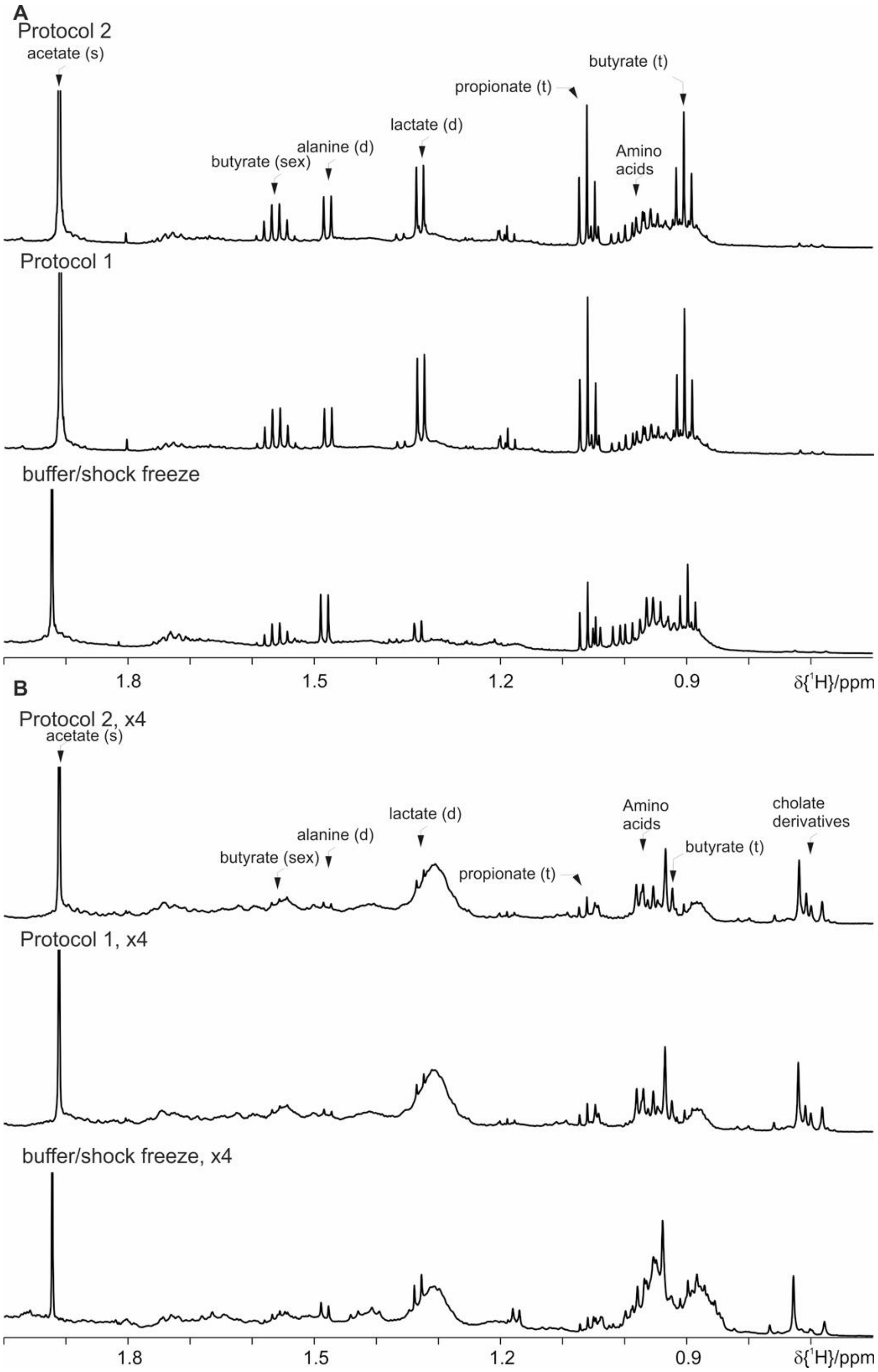

Figure S6**.** Comparison of Protocol 1 and 2 with the basic protocol with shock freeze: (**A**) feces of mice fed on a standard diet and (**B**) on high fat diet (HFD) (the intensity is upscaled four times). For clarity only a selected spectral range (0.6 ppm till 2.0 ppm) of the proton NMR spectra is shown and the most prominent metabolites are assigned. Protocol 1 and 2 clearly extract more efficiently the SCFA, based on their increased intensity for both diets.

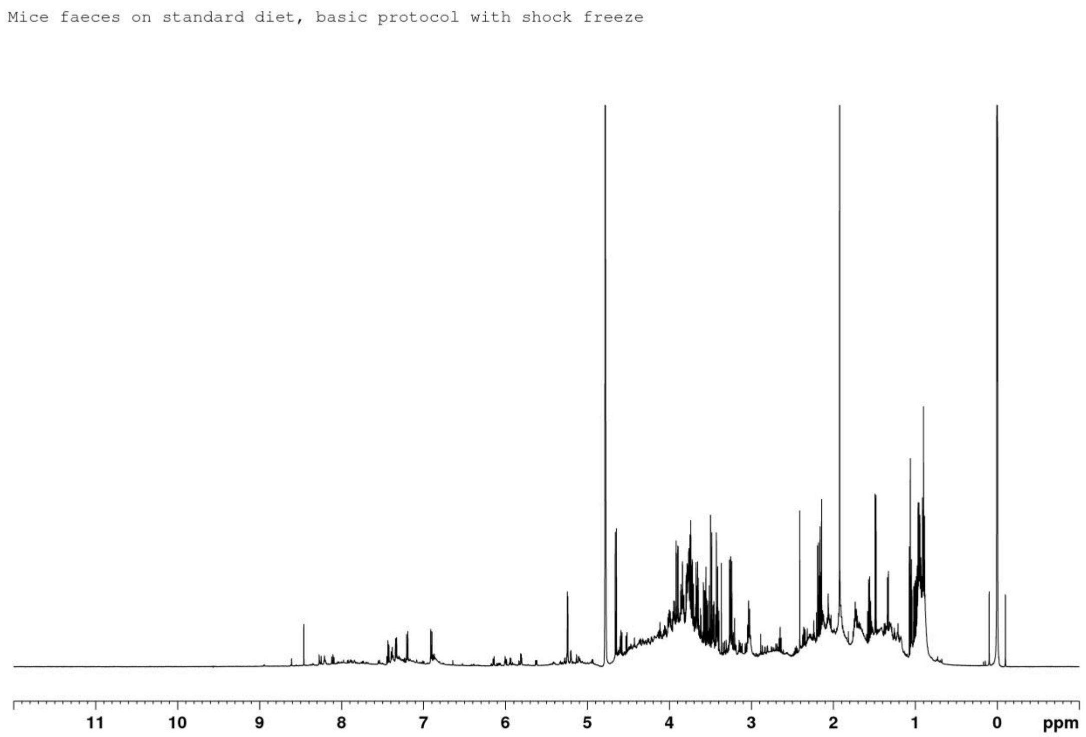

Figure S7. 1H NMR spectrum of the extracted metabolites from mice on a standard diet with phosphate buffer protocol with shock freeze

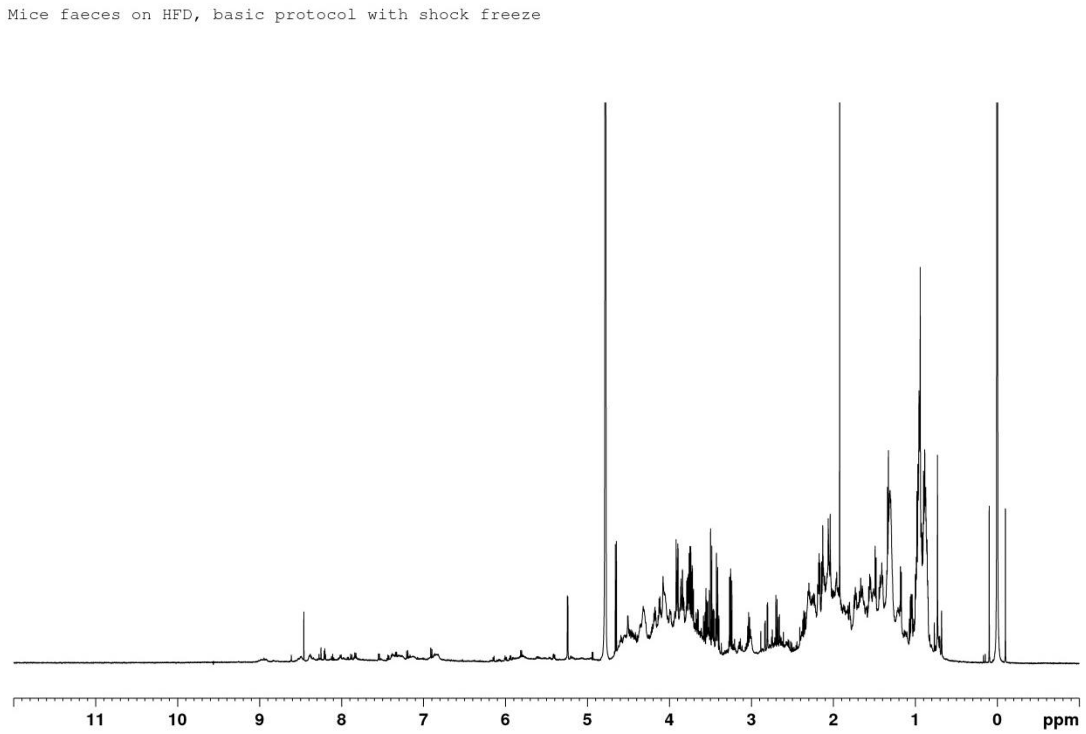

Figure S8. 1H NMR spectrum of the extracted metabolites from mice on a HFD with phosphate buffer protocol with shock freeze

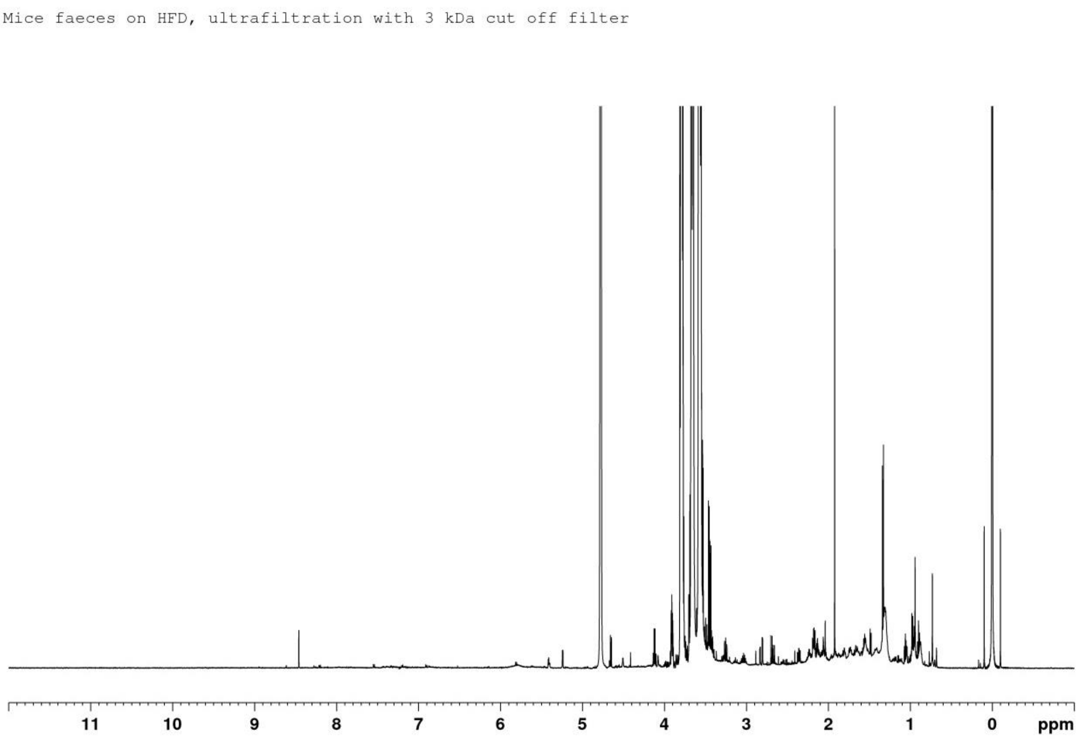

Figure S9. 1H NMR spectrum of the extracted metabolites from mice on a HFD with ultrafiltration (3kDa cut off filter)

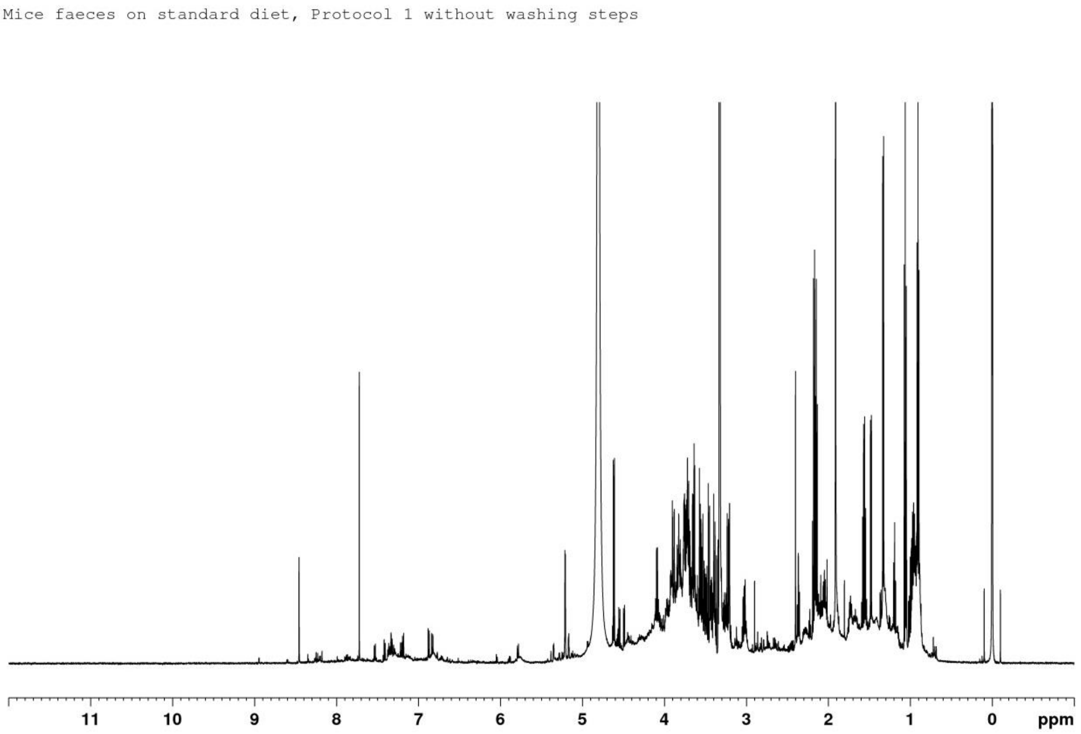

Figure S10. 1H NMR spectrum of the extracted metabolites from mice on a standard diet with Protocol 1 without washing steps.

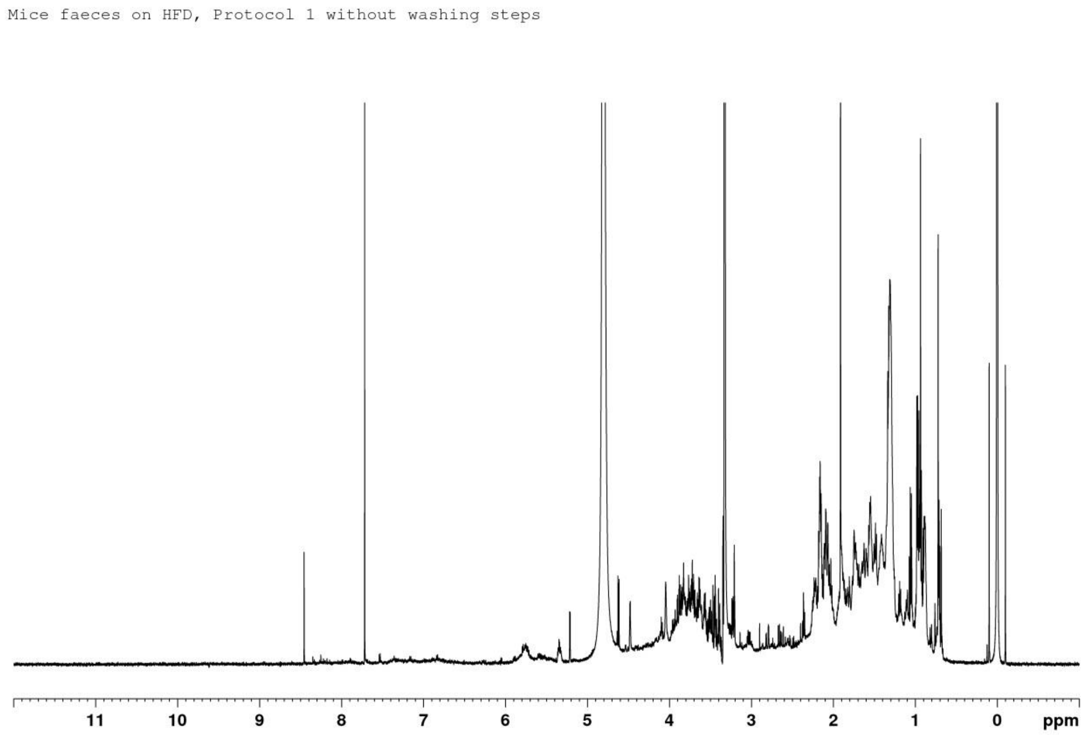

Figure S11. 1H NMR spectrum of the extracted metabolites from mice on a HFD with Protocol 1 without washing steps.

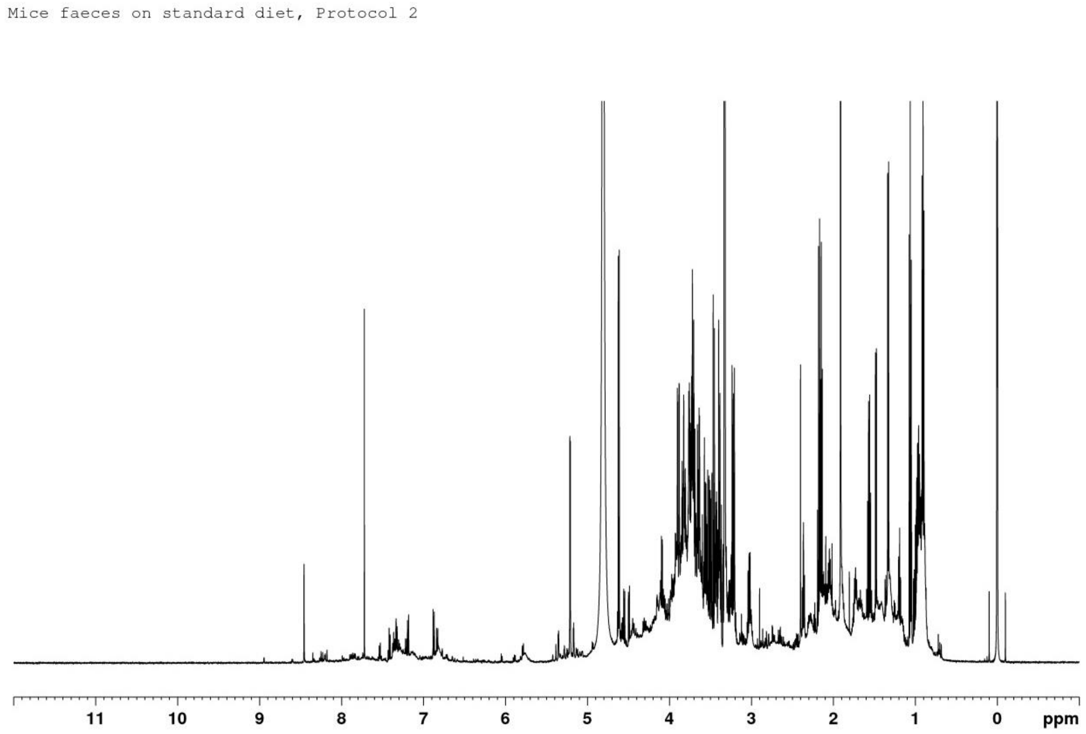

Figure S12. 1H NMR spectrum of the extracted metabolites from mice on a standard diet with Protocol 2.

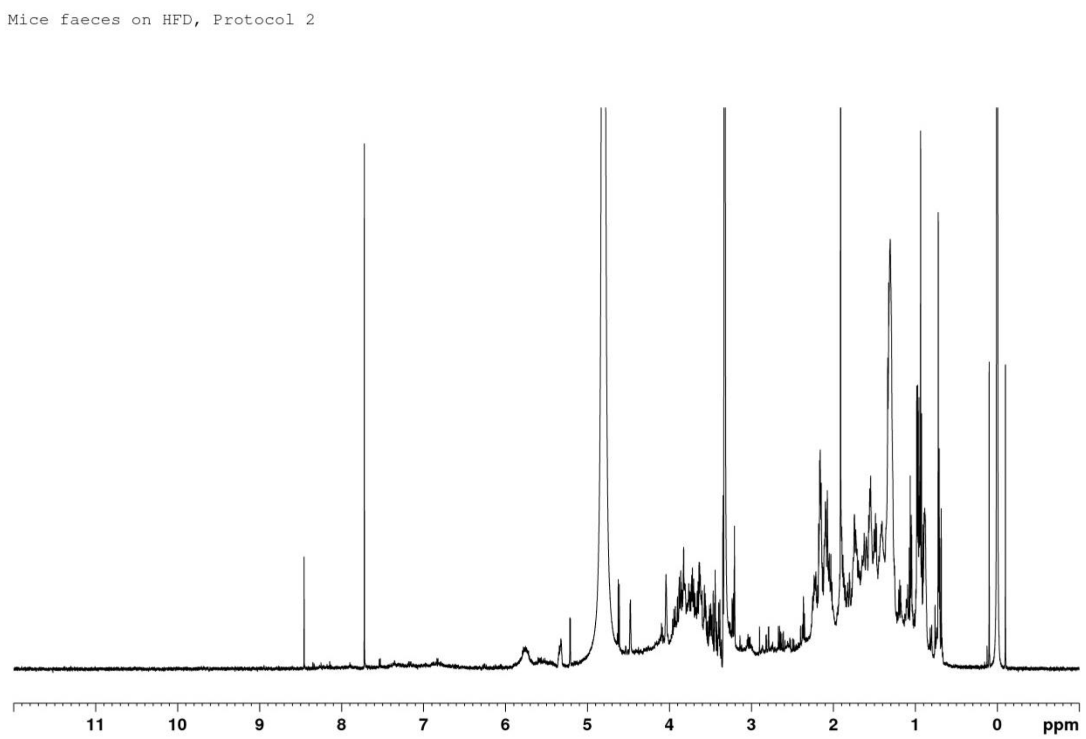

Figure S13. 1H NMR spectrum of the extracted metabolites from mice on HFD with Protocol 2.

References

1. Wu, J.; An, Y.; Yao, J.; Wang, Y.; Tang, H. An optimised sample preparation method for NMR-based faecal metabonomic analysis. *Analyst* **2010**, *135*, 1023–1030, doi:10.1039/b927543f.
2. Lamichhane, S.; Yde, C.C.; Schmedes, M.S.; Jensen, H.M.; Meier, S.; Bertram, H.C. Strategy for Nuclear-Magnetic-Resonance-Based Metabolomics of Human Feces. *Anal. Chem.* **2015**, *87*, 5930–5937, doi:10.1021/acs.analchem.5b00977.
3. Daniel Kraus; Qin Yang; Barbara B. Kahn. Lipid Extraction from Mouse Feces. *Bio. Protoc.* **2015**, 1–5, doi:10.21769/BioProtoc.1375.
